# Elders Health Empowerment Scale: Spanish adaptation and psychometric analysis

**Published:** 2014-12-30

**Authors:** Daniel Jorge Luis Serrani Azcurra

**Affiliations:** Professor at Department of Biological Sciences, Faculty of Psychology, National University of Rosario, Rosario, Argentina; Maimónides University, Buenos Aires, Argentina

**Keywords:** Health, empowerment, rating scale, elders, chronic disease, psychometric analysis, self-efficacy

## Abstract

**Introduction:**

Empowerment refers to patient skills that allow them to become primary decision-makers in control of daily self-management of health problems. As important the concept as it is, particularly for elders with chronic diseases, few available instruments have been validated for use with Spanish speaking people.

**Objective:**

Translate and adapt the Health Empowerment Scale (HES) for a Spanish-speaking older adults sample and perform its psychometric validation.

**Methods:**

The HES was adapted based on the Diabetes Empowerment Scale-Short Form. Where "diabetes" was mentioned in the original tool, it was replaced with "health" terms to cover all kinds of conditions that could affect health empowerment. Statistical and Psychometric Analyses were conducted on 648 urban-dwelling seniors.

**Results:**

The HES had an acceptable internal consistency with a Cronbach's α of 0.89. The convergent validity was supported by significant Pearson's Coefficient correlations between the HES total and item scores and the General Self Efficacy Scale (r= 0.77), Swedish Rheumatic Disease Empowerment Scale (r= 0.69) and Making Decisions Empowerment Scale (r= 0.70). Construct validity was evaluated using item analysis, half-split test and corrected item to total correlation coefficients; with good internal consistency (α> 0.8). The content validity was supported by Scale and Item Content Validity Index of 0.98 and 1.0, respectively.

**Conclusions:**

HES had acceptable face validity and reliability coefficients; which added to its ease administration and users' unbiased comprehension, could set it as a suitable tool in evaluating elder's outpatient empowerment-based medical education programs.

## Abstract (es)

**Introducción:**

Empoderamiento se refiere a las habilidades que le permiten al paciente convertirse en responsable de tomar las decisiones para el control diario de sus problemas de salud. A pesar de ser un concepto tan importante, particularmente para adultos mayores con problemas crónicos de salud, hay pocos instrumentos accesibles que hayan sido validados para su uso en hispano-hablantes.

**Objetivos:**

Traducir y adaptar la Escala de Empoderamiento sobre la Salud (EES) para una muestra de adultos mayores hispano-hablantes y llevar a cabo su validación psicométrica.

**Métodos:**

La EES se adaptó basándose en la Escala de Empoderamiento de la Diabetes versión corta. Donde se mencionaba "diabetes" en el instrumento original, se reemplazó con el término "salud" para cubrir todos los tipos de condiciones que podrían afectar el Empoderamiento sobre la salud. Se realizaron análisis estadísticos y psicométricos sobre 648 adultos mayores residentes urbanos.

**Resultados:**

La EES tuvo una consistencia interna aceptable con un α de Cronbach de 0.89. La validez convergente se apoyó en un coeficiente de correlación de Pearson significativo entre la EES total y por ítems y la Escala General de Auto-eficacia (r= 0.77), la Escala de Empoderamiento para la Enfermedad Reumática versión Sueca (r= 0.69) y la Escala de Empoderamiento Tomando Decisiones (r= 0.70). La validez de Constructo se evaluó mediante análisis de ítem, test de las dos mitades y coeficiente de correlación ítem corregido total (α > 0.8). La validez de contenido se apoyó por los Índices de Validez de Contenido para la Escala y para los ítems de 0.98 y 1.0, respectivamente.

**Conclusiones:**

La EES tuvo una validez y confiabilidad aceptables, que sumados a su facilidad de administración y comprensión simple y sin sesgos podría constituirse en una herramienta confiable para evaluar programas educativos médicos basados en el Empoderamiento de pacientes mayores ambulatorios.

## Introduction

The medical health literature on empowerment has increased exponentially since the early 1990s, particularly in relation to chronic conditions 1. Empowerment is a workable and patient-centered approach leading to effective interventions for addressing the psychosocial components of living with chronic diseases. In order to accomplish those goals, it must be recognized that patients are the primary decision-makers in control of the daily self-management of their health problems, and emphasizing patient autonomy and expansion of freedom of choice is mandatory 2. The concept of empowerment is deeply rooted in social sciences 3 and has been defined as the complex and multifaceted process of recognizing self needs, skills and resources, improve owns abilities to solve problems, reach a sense of power or control and enable people gain mastery over their lives 4. This concept is being included as part of the usual health practices in modern medicine occurring within the context of a nurturing physician-client relationship 5. It is expected that through this process of empowerment, client's perceptions of competence regarding the ability to maintain good health and manage interactions with the health care system would improve, as a result of the internalization of current health ideas and goals at the individual and societal level. In the case of older adults, empowerment should promote well-being, healthy lifestyles and social connectedness 6. According to those trends, interventions have been actively developed based on empowerment theory 7 and various instruments have been in use to acknowledge different chronic medical conditions ranging from rheumatic diseases 8 to cardiovascular illness or diabetes 9. The Diabetes Empowerment Scale-short form (DES-SF) 10 measures empowerment in patients with diabetes, has been tested with elder patients and has been adapted to evaluate health-related empowerment. However, a Spanish translation or adaptation is lacking, so the goals of this study were to translate and adapt the DES-SF as a Health Empowerment Scale for Spanish-speaking elders, and assess its psychometric properties. 

## Materials and Methods 

### Instruments

The Health Empowerment Scale (HES) was adapted from The Diabetes Empowerment Scale Short-form (DES-SF), which was selected for its brevity, reducing the chance of non-response due to poor concentration, and its excellent validity and reliability criteria, while reflecting the attributes of empowerment. The mean age of subjects in the original data set and follow-up studies was 60 years, which further supports its use for elderly subjects. After substituting the word "diabetes" with "health" in each item from the DES-SF to assess health-related empowerment, the instrument retained 8 items, scored on a 5 points Likert scale ranging from 5 (strongly agree) to 1 (strongly disagree). Higher scores indicate stronger level of health-related empowerment (Table 1).

**Table 1.**
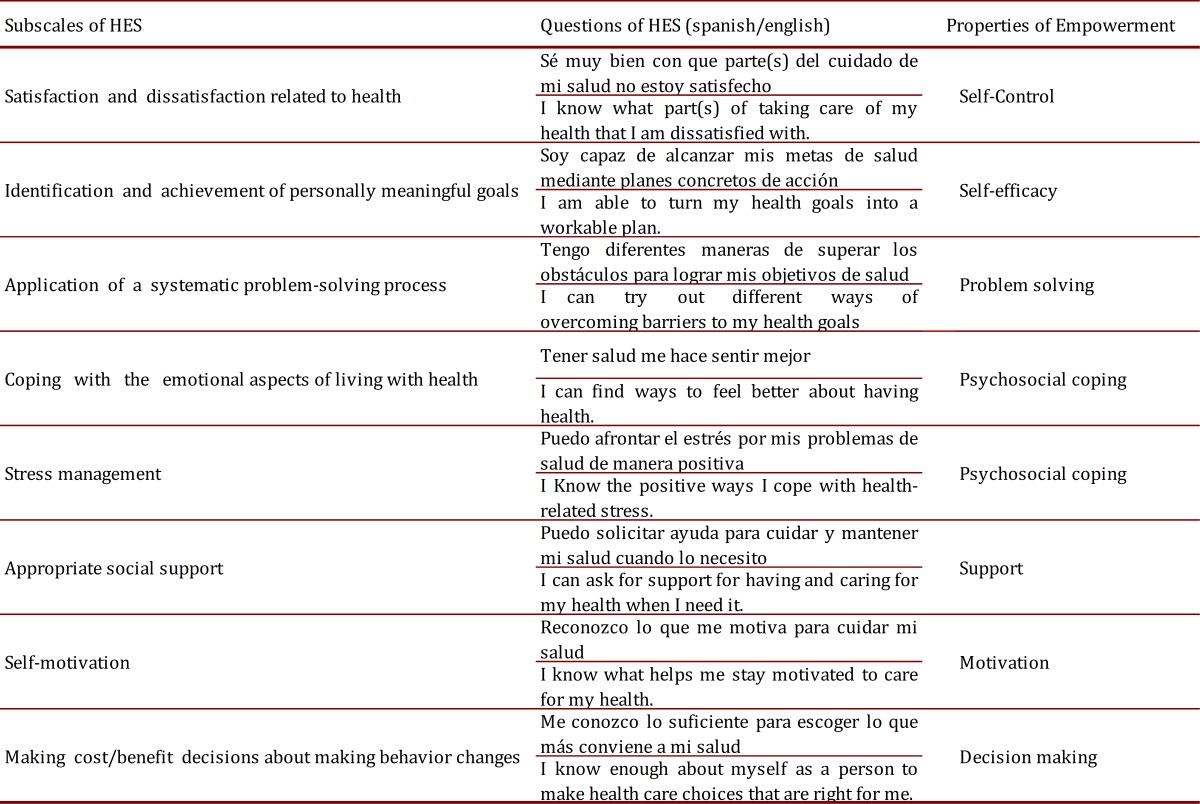
Relationship between HES and properties of empowerment.

Other related instruments used for concurrent validity were: 

Making Decisions Empowerment Scale 11 is a 28-item self report questionnaire designed to measure empowerment in patients with psychological disorders; with five subscales (self-efficacy, power, community activism, righteous anger and optimism over the future) responded to on a four-point scale, exhibiting good internal consistency (Cronbach's alpha = 0.81). 

Swedish Rheumatic Disease Empowerment Scale (SWE-RES-23) 8 adapted for use in patients with rheumatic diseases, has 23 items responded on a 4-points Likert scale, containing five factors (empowerment subscales), and a Cronbach's α = 0.59 to 0.92.

The General Self Efficacy Scale (Spanish Adaptation) 12 assess people's believe about their ability to appropriately manage life stressors. It has 10 items rated with a 10 point Likert scale and has high internal consistency (between 0.87) and predictive value with other correlated scales.

### Procedures 

To assess the validity and reliability of the Health Empowerment Scale (HES), the DES-SF was translated into Spanish by a certified translator and two physicians specialized in gerontology. Each time the word "diabetes" appeared, it was replaced by the word "health" to accomplish the general purpose of the instrument. Another certified translator, blind to the first translation, translated the HES back into English. After that, a content analysis was performed by two gerontology professors and one licensed nurse with training in gerontology. They were asked to make comments on individual questions in relation to the accuracy, clarity, cultural relevance semantic, conceptual and operational equivalence of the translation 13. Questions number 4 and 8 were evaluated as difficult to understand directly (regarding fluency) and were rephrased, and question 6 mixed the terms "health care" and "health". All questions were edited in light of the comments. A pilot study (n= 32) was performed with the panel-modified version at a community senior center on August 2013. The researcher read the scale in a Spanish consistent manner and recorded the responses. The HES required an average of 18 min to complete. The panel-modified version was readily accepted. Furthermore, to assess its readability properties the scale was processed with the INFLESZ^®^ 1.0 software which issued a legibility index of 69.87 (acceptable when ≥55) meaning that the scale was considered simple to understand according to an elementary literacy level 14. Therefore, in September 2013, the main study was conducted for 700 urban-dwellers senior citizens assisting to community day-centers located in Rosario (Argentina). The data were collected by three nursing majors who had been trained to perform the survey in a similar and constant manner. They administered the questionnaire one-on-one. Of a total of 700 copies distributed for the survey, 648 were completed and returned.

### Ethical considerations

The study was carried out after receiving approval from the institutional review board at National University of Rosario. Written informed consents were obtained when the participants agreed to participate in the study.

### Sample

Subjects were randomly selected from a large population of senior citizens regularly assisting to community day-centers located in Rosario. A sample size of 103 subjects was required in order to get an effect size of 0.3, a significance level of 0.01 for a type I error and a power of 0.8 at correlation analysis 15. Below is the formula used to calculate the sample size, according to Altman's 16.

However, taking into account number of participants to figure out sample size in factor analysis, some authors 17 have suggested the following guide samples sizes: 50 as very poor; 100 as poor, 200 as fair, 300 as good, 500 as very good and 1,000 as excellent. According to that, we decided to include in the final sample 648 elderly individuals.

### Statistical analysis

Descriptive statistics were used to establish the frequency, range, mean, and SD of demographic and clinical characteristics of the main sample. The HES reliability was assessed through internal consistency with total Cronbach's alpha, half-split analysis, Item-to-total correlations and Cronbach's alpha without item. Validity was evaluated through Principal and Confirmatory Factor Analysis (maximum likelihood). The SPSS^®^ statistical package for the Social Sciences (SPSS^®^-19) was used to compute descriptive statistics, correlation and internal consistency together with t-test analyses. Principal component analysis allowed performing statistical significance testing of factor loadings, correlations among factors and computation of confidence intervals used for factor extraction. After extraction, factors were retained for rotation according to eigenvalues greater than 1.0, items loadings above 0.30, no or few item cross-loadings and no factors with fewer than three items. Varimax was the selected orthogonal rotation method as it assumes no correlation exists between factors, rendering a more accurate solution. Factorability of the correlation matrix was based on Bartlett's test of sphericity to estimate the probability that correlations in a matrix were 0, and the Kaiser-Meyer-Olkin (KMO) measure of sampling adequacy which accounts for the relationship of partial correlations to the sum of squared correlations, thus indicating the extent to which a correlation matrix actually contains factors or simply chance correlations between a small subset of variables. Values ≥0.60 are required for good factor analysis. Confirmatory factor analysis (CFA) was carried out using the maximum likelihood (ML) method. Since departures from multivariate normality can have a significant impact on maximum-likelihood estimation, we calculated descriptive analytical measures prior to conducting CFA analysis. As kurtosis statistics were found to indicate normality, no other correction was used to adjust the model chi-square 18. According to Schweizer's recommendations 19 additional measures of model fit were used: root mean square error of approximation (RMSEA) with values below 0.08 considered acceptable fit; comparative fit index (CFI) ≤0.06 as good fit values; GFI and NNFI with values ≥0.90 as acceptable fit. Three putative models were compared with one, two and three factors. The last (three factor) was a less parsimonious model, in which the factor inter-correlation was freely estimated; while the first model (one factor) was a more parsimonious model, the independence model, in which the factor inter-correlation was set to 0 to represent the factor structure of the original scale. Concurrent validity was tested with Pearson correlation between HES and GSES, SRDES and MDES. Content Validity was evaluated with Scale and Item Construct Validity Index. Statistical significance was established at *p*= 0.01 (two-tails). 

## Results 

Participants' socio-demographic data are displayed in Table 2. Most of them were married females, with a mean age of 75.5 for men and 74.1 for women, ranging from 64 to 93 years; most of them female (71.0%), and most (89.7%) had one or more chronic diseases, two-thirds with hypertension, nearly half of them with arthritis and one-third with diabetes. On average they had 2.21 chronic diseases / person and took an average of 2.86 types of different drugs. Most participants achieved incomplete literacy level, had regular medical consultation, but rarely had been admitted to hospital. They had medium incomes level and exhibited mild self-efficacy scores. A summary of the baseline characteristics of participants is offered in Table 2.

**Table 2.**
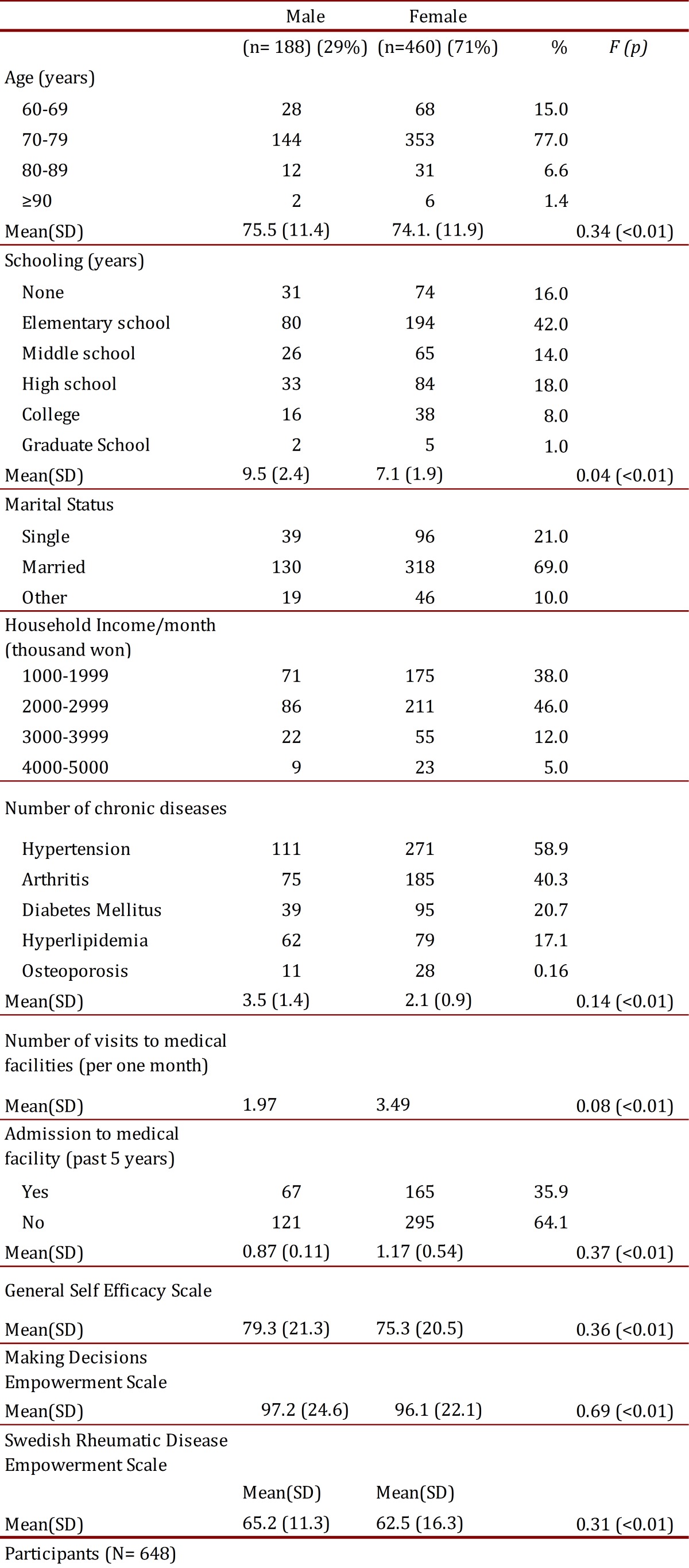
Demographic characteristics of subject (n= 648).

### Reliability 

The mean HES score for the sample was 3.5 (SD 0.73), every question was in the range between 3.22-3.79 (SD 1.03-1.22). Coefficient of kurtosis (-0.764 to 0.077) and skewness (1.843-3.058) showed a normal distribution. HES exhibited excellent internal consistency with Cronbach's α= 0.89 and >0.81 for full scale and corrected items to total correlation respectively. Floor and ceiling effects were small (<20%), suggesting that HES has significant power to measure health empowerment level of older adults (Table 3). Test-retest reliability was assessed in 23 participants evaluated by the same researcher with an interval of 3 months, and intra-class correlation coefficient (ICC) was 0.92 (*p*= 0.001) suggesting good stability along time. With the half-splitting analysis of the scale, first and second halves showed a good reliability coefficient of 0.86 and 0.91 respectively.

**Table 3.**
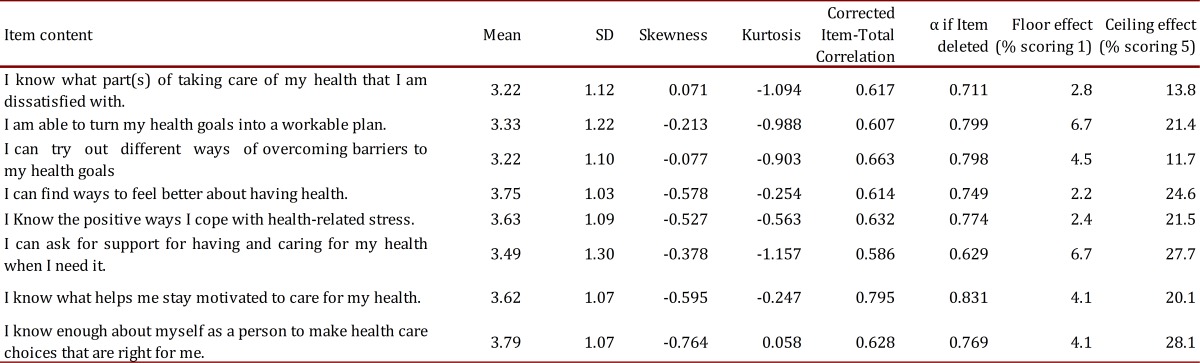
Statistics, Corrected Item-Total Correlation, Floor-ceiling effects for HES (n= 648).

### Validity

Construct validity was demonstrated by Kaiser-Meyer-Olkin sampling (KMO = 0.890) and Bartlett's test of sphericity (χ^2^_(634)_= 5425.72; *p*< 0.001) which showed that sample size was suitable for conducting factor analysis (least factor load method) and the correlation matrix had not occurred by chance. A single factor solution was judged the more acceptable, explaining 52.4% of the variance. Confirmatory Factor Analysis on three putative models (with one, two and three factors) showed the best fitness index for the one factor scale with CFI, GFI and NNFI ≥0.90, and RMSEA ≤0.06 as good fit values (Table 4) 

**Table 4.**
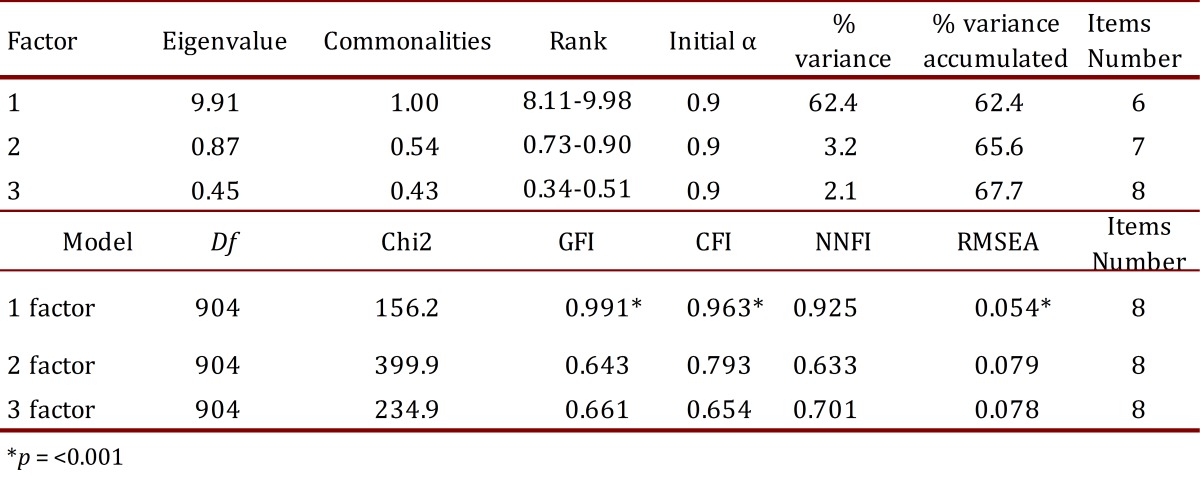
Psychometric results from exploratory and confirmatory factor analysis.

### Concurrent validity

Significant Pearson's correlations were found between Total and Items' HES scores and the other three scales (GSES, SRDES and MDES) (Table 5).

**Table 5.**
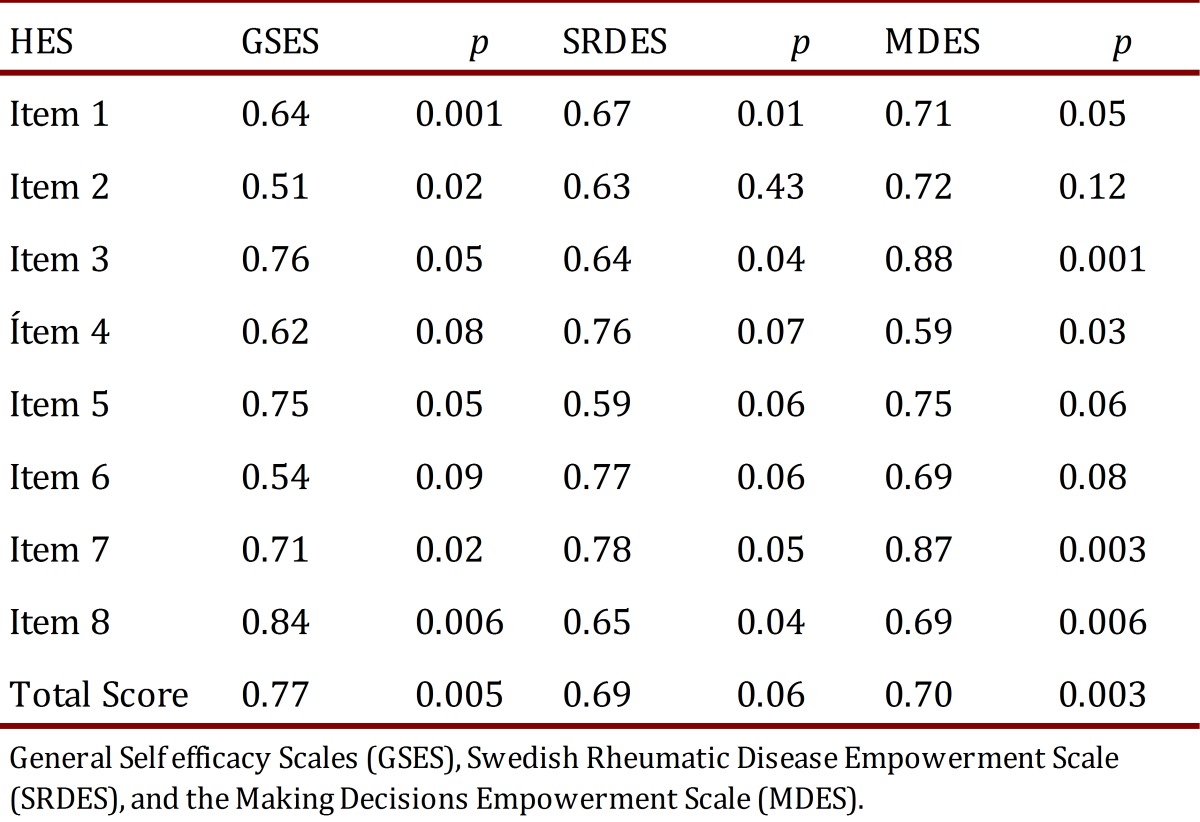
Coefficient correlations between the 8 items of the HES and the other scales.

### Content validity

After the survey, three gerontologists, two licensed nurses with gerontology training, and the author rated each question as a valid measure of the construct using a five-point Likert scale (1= strongly disagree, 5= strongly agree). Based on their responses, an item (I-CVI) and scale (S-CVI) content validity index scores of 0.98 and 1.0 respectively, were accepted as high.

## Discussion

In conclusion, the HES showed excellent reliability and validity for assessing the health-related empowerment of elderly individuals. First, the mean of the HES was 3.51, which shows that senior citizens have an empowerment level above medium on a score of 3, which represents "neither agree nor disagree" on the Likert scale. The same results were found in other studies using similar instruments such as the Empowerment for Self Care Scale 20 (Mean=3.65, SD=0.40), or the Patient Empowerment Scale (Mean=3.68, SD=0.53) 21. Both the floor and ceiling effects were small according to usual criteria 22. The ceiling effect refers to patients who start with higher empowerment abilities than the average patient and lack room for improvement, while floor effect means the opposite as these subjects have lower empowerment skills than average and so may show greater (and biased) improvements. As a consequence, HES was balanced enough to correctly measure health empowerment interventions outcomes without skewing the end results. HES Cronbach's α was good (0.89) compared to DES-SF reliability (0.84). Corrected item-total correlation was high for all questions (>0.81) although the lower value was for question 6. A possible explanation could be that participants regarded supporters to be acquaintances such as family members or friends, and many elders reported that they should not ask their family for help because they did not want to be a burden for their family. Instead they build on the sense that empowerment should render them independent from their family. So item 6 content should add health care providers or health care system to family and friends as putative support providers. Content validity of the HES was acceptable (I-CVIs= 1.0, S-CVI= 0.98). Construct validity was supported by significant Pearson correlations between the HES total scores and the SRDES (*r*= 0.69), GSES (*r*= 0.77) and MDES (*r*= 0.70). This result shows that there is a strong correlation between empowerment and self-efficacy, supported by previous researches 6, confirming that self-efficacy is both a component and a result of empowerment 23. It has been argued that health profile of the future elderly population in Latin America will be less predictable due to factors associated with demographic past that haunt them for a long time and make them more vulnerable, even if economic and institutional conditions turn out to be better than what they are likely to be 24. The number of chronic conditions will probably increase with age and would be higher among females than among males; levels of self-reported diabetes and obesity will be higher than those found in the US; along with more deteriorated health and functional status in the region. According to these assertions, it seems a valuable strategy to strengthen elder citizens' health promotion activities in our region as a way to reduce inequalities in health care access and improve chronic diseases outcomes such as diabetes or obesity 25. Although exhibiting its usefulness in measuring empowerment, several questions must be further developed and tested. Some limitations that can be endowed to this research are that this instrument measures empowerment skills at an individual level, excluding the organizational and community ones, which may be important in the case of the frail or disabled elder. Another limitation that should be considered is that, despite the fact that western cultures share common tenets, sometimes subtle differences between them may account for some issues like sentence comprehension or resource availability which could make the questionnaire not directly suitable in Latin America culture, highlighting the need to take cultural differences between countries into account when adapting questionnaires. It may be suitable to perform a predictive assessment, evaluating participants' health one year later and comparing it with the present health status.

##  Conclusion

The HES scale possesses acceptable validity and reliability. Considering its brevity and ease of administration, the HES can be used as an outcome measure for the empowerment of Spanish-speaking senior citizens. 
